# Impact of the COVID-19 Pandemic on Surgical Treatment of Patients With Urological Diseases at a University Hospital

**DOI:** 10.7759/cureus.29572

**Published:** 2022-09-25

**Authors:** Matheus P Mariani, Fernando Nestor Facio, Luís Cesar F Spessoto

**Affiliations:** 1 Surgery, Medical School of São José do Rio Preto (FAMERP), São José do Rio Preto, BRA

**Keywords:** public health, urology, treatment, surgery, coronavirus

## Abstract

Background: The coronavirus disease 2019 (COVID-19) appeared in China and spread quickly to other regions of the country and around the world, changing the way of life of individuals and the routine of healthcare systems.

Objective: The aim of the present study was to investigate the impact of the pandemic on the surgical treatment of patients with urological diseases at a university hospital.

Materials and methods: A retrospective analysis of the charts of patients with urological diseases submitted to surgical treatment between January 2019 and December 2020 was conducted. The variables of interest were age, sex, and most performed surgical procedures (double-J stent placement, cystoscopy, vasectomy, removal of double-J stent, ureterolithotripsy, endoscopic bladder procedure, kidney transplant, and endoscopic prostate procedure).

Results: Around 59.03% of patients with urological diseases who had surgery in 2019 were male; placement of the double-J stent accounted for 35.85% of all surgeries; 3556 surgical procedures were performed. In 2020, 57.22% of the patients were male, placement of the double-J stent accounted for 38.34% of all surgeries, and 3093 surgical procedures took place. Analyzing the types of surgery conducted in 2019 and 2020, a significant reduction occurred in the number of procedures in 2020 (p = 0.000).

Conclusion: The pandemic exerted an impact on the surgical treatment of patients with urological diseases at a university hospital. No significant changes occurred with regard to the sex and age of the patients, but a significant difference was found in the number of surgical procedures performed.

## Introduction

At the end of 2019, COVID-19 emerged in China and spread throughout the world [1-3], changing the way of life of its population as well as the routine of healthcare providers. Changes also occurred in the field of urology, with effects exerted on the number of appointments, elective surgeries, emergency surgeries and the number of hours worked [4-7].

With the situation imposed by the pandemic, guidelines were created to assist urologists in daily practice, including discussions on the priority of procedures i.e., which procedures can/cannot be delayed and how to perform such procedures [8-11]. An analysis of the literature reveals that the focus of studies was on changes in surgeries and the routine of urologists, with new guidelines for urological practice. However, studies on patients affected by the pandemic are scarce. No investigations were conducted to determine the patients who had their surgical procedures delayed, the preference for noninvasive treatments, and the reduction in the number of surgeries performed.

The present study aimed to investigate the impact of the pandemic on the surgical treatment of patients with urological diseases at a university hospital.

## Materials and methods

A retrospective, descriptive, cross-sectional study was conducted involving the surgeries performed by the urology service in pediatric and adult patients between January 2019 and December 2020 and recorded in the databases of the hospital. This study was conducted at the public hospital affiliated with the São José do Rio Preto School of Medicine and received approval from the local institutional review board (certificate number: 45760021.4.0000.5415).

The most performed procedures at the hospital were considered, such as the placement of the double-J stent, cystoscopy, vasectomy, removal of the double-J stent, and ureterolithotripsy. Procedures that the authors judged as having clinical relevance were also analyzed, such as endoscopic bladder procedures, endoscopic prostate procedures, and surgeries related to kidney transplantation (unilateral or bilateral kidney removal from a deceased donor, transplantation of kidney from a living donor, transplantation of kidney from a deceased donor and kidney received by a patient hospitalized with a private health insurance plan).

The following variables of interest were also collected to characterize patients who potentially remained without surgical treatment due to the pandemic: sex (male or female), age of patients who underwent procedures in 2019 and 2020, type of procedure (listed above), and quantity of procedures performed in the pre-pandemic year and the first year of the pandemic.

The data were entered into Excel spreadsheets (Microsoft Corp., Redmond, Washington, USA). The descriptive analysis involved the calculation of central tendency and dispersion measures and frequencies. The Kolmogorov-Smirnov test was used to determine the normality of the data. The Mann-Whitney test and Pearson’s chi-squared test were used for the comparison of frequencies. The Statistical Package for Social Sciences (SPSS), version 23 (IBM Corp., Armonk, NY, USA) and GraphPad InStat, version 3.10 (GraphPad Software, San Diego, CA, USA) were used for the analyses, with a p-value ≤ 0.05 considered indicative of statistical significance.

## Results

Among all patients with urological diseases who submitted to surgical treatment in 2019, 59.03% were male; the placement of the double-J stent accounted for 35.85% of all surgeries; a total of 3556 surgeries were performed considering the procedures studied. In 2020, 57.22% of the patients were male, placement of the double-J stent accounted for 38.34% of all surgeries and a total of 3093 surgical procedures were performed (Table 1). No significant difference in age group was found between 2019 and 2020 (p = 0.0669, Mann-Whitney test).

**Table 1:** Distribution of variables in patients with urological diseases who underwent surgical treatment at a university hospital in 2019 and 2020

Variables	2019		2020	
	N	%	N	%
Sex				
Male	2099	59.03	1770	40.97
Female	1457	57.22	1323	42.78
Type of surgery				
Double-J implant	1275	35.85	1186	38.34
Cystoscopy	1024	28.79	972	31.42
Vasectomy	355	9.98	166	5.36
Double-J removal	297	8.35	199	6.43
Ureterolithotripsy	289	8.12	297	9.60
Endoscopic bladder procedure	136	3.82	139	4.49
Kidney transplant	134	3.76	82	2.65
Endoscopic prostate procedure	46	1.29	52	1.68

No significant difference was found in the sex of patients with urological diseases who underwent surgical treatment in 2019 and 2020 (p = 0.138, Pearson’s chi-squared test). Analyzing the types of surgery performed in 2019 and 2020, a significant reduction occurred in the number of procedures in 2020 (p = 0.000) (Table 2).

**Table 2:** Comparison of results related to patients with urological diseases who underwent surgical treatment in 2019 and 2020 * significant difference, Pearson’s chi-squared test

Variables	2019	2020	p-value
Sex			
Male	2241	1875	0.138
Female	1596	1440	
Type of surgery			
Double-J implant	1275	1186	
Cystoscopy	1024	972	
Vasectomy	355	166	
Double-J removal	297	199	0.000*
Ureterolithotripsy	289	297	
Endoscopic bladder procedure	136	139	
Kidney transplant	134	82	
Endoscopic prostate procedure	46	52	

## Discussion

The results of the present study demonstrated a significant impact of the pandemic on the surgical treatment of patients with urological diseases at a tertiary university hospital. Comparing the years 2019 and 2020, the number of procedures for the majority of surgeries (placement of the double-J stent, cystoscopy, vasectomy, removal of double-J stent, and kidney transplant) was significantly lower in the year of the pandemic. The exceptions were endoscopic bladder procedures, ureterolithotripsy, and endoscopic prostate procedures. This may be explained by the fact that such procedures are employed in situations of urological urgency and in cases of cancer treatment, as no changes in care had occurred for these clinical conditions [12].

The greatest reductions in the year of the pandemic were related to elective, non-oncological surgeries, such as vasectomy and kidney transplantation, due to the possibility of rescheduling and awaiting a more favorable scenario. Despite the importance of kidney transplantation for patients with chronic kidney failure, a drastic reduction in the quantity of these procedures was found in 2020 at the tertiary hospital, which is a regional reference center for the population.

A significant reduction was also found in the number of procedures performed for the removal of the double-J stent. However, this may have been due to the failure of patients to return to the hospital for removal of the stent after the minimum period of three months. Forgetfulness may also have occurred, as patients may have been asymptomatic, which, together with the social isolation imposed by the pandemic, may have contributed to this reduction.

In the scenario of the pandemic, uro-oncological surgeries should be maintained at tertiary centers, which ideally maintain the capacity to deal with the complications of COVID-19 and provide safe care for cancer patients [13,14]. This explains why the number of procedures related to oncological treatment either did not diminish or the reduction was non-significant, contrasting with the decrease in elective procedures. However, follow-up of these uro-oncological patients is necessary. Due to the pandemic, some conditions, such as prostate cancer, may have progressed to metastasis due to the more distant follow-up and delayed surgeries [13]. Similarly, the number of urgent surgeries diminished less in comparison to elective procedures. This may have occurred because the present study was conducted at a reference hospital for urgent and emergency care in the region, which followed the guidelines issued by urological societies for conduct during the pandemic [8,12].

The limitation of this study is that the research is based on a retrospective study performed at a single medical center. Long-term and multi-centric studies are necessary to corroborate our findings.

## Conclusions

In this investigation, the analysis of the impact of COVID-19 pandemic on surgical treatment of patients with urological diseases at a tertiary university hospital revealed no significant changes occurred with regards to the sex and age of the patients. Moreover, the quantity of procedures for the majority of surgeries including placement of double-J stent, cystoscopy, vasectomy, removal of double-J stent, and kidney transplant was significantly lower in the year of the pandemic.
